# A guided participation nursing intervention to theraupeutic positioning and care (GP_Posit) for mothers of preterm infants: protocol of a pilot randomized controlled trial

**DOI:** 10.1186/s40814-020-00601-5

**Published:** 2020-05-26

**Authors:** Andréane Lavallée, Marilyn Aita, José Côté, Linda Bell, Thuy Mai Luu

**Affiliations:** 1 Faculty of Nursing, Université de Montréal, Montréal, Canada; 2 CHU Sainte-Justine Research Centre, Montréal, Canada; 3 Quebec Network on Nursing Intervention Research (RRISIQ), Quebec, Canada; 4 Montreal University Health Center (CHUM) Research Center, Montréal, Canada; 5 School of Nursing, Faculty of Medicine and Health Sciences, Univertisé de Sherbrooke, Sherbrooke, Canada; 6 Department of Pediatrics, CHU Sainte-Justine, Montréal, Canada; 7 Department of Pediatrics, Université de Montréal, Montreal, Canada

**Keywords:** Guided participation, Maternal sensitivity, Neonatal intensive care unit, Neurodevelopment, Preterm

## Abstract

**Background:**

In the NICU, interventions intended to enhance maternal sensitivity are indicated in order to optimize preterm infant development and long-term mother-infant attachment. A novel nursing intervention was developed following a theory-oriented methodology and is based upon the guided participation theory for mothers to participate in their preterm infant’s therapeutic POSITioning and care (GP_Posit). The primary objective of this study is to evaluate the feasibility and acceptability of (i) the study design; and (ii) the experimental GP_Posit nursing intervention during NICU hospitalization. The secondary objective is to estimate the preliminary effects of GP_Posit on maternal and preterm infant outcomes.

**Methods:**

A pilot parallel-group randomized clinical trial (RCT) was designed where mother-preterm infant dyads are being recruited and randomized to a control group (usual care) or experimental group (GP_Posit intervention). Data collection includes feasibility and acceptability data as well as preliminary effects on maternal sensitivity and infant neurodevelopment. Ethical approval from the University Hospital ethical board was obtained in January 2018 (2017–1540).

**Discussion:**

Data collection for this pilot study is expected to end in 2020. Results of this pilot study will inform about the feasibility and acceptability of the study design and GP_Posit intervention, a nursing intervention having the potential to favor maternal sensitivity and infant neurodevelopment in the NICU and guide the elaboration of a large-scale RCT.

**Trial registration:**

clinicaltrial.gov, NCT03677752. Registered 19 September 2018.

## Introduction

### Background and rationale

While in the neonatal intensive care unit (NICU), the preterm infant’s brain requires nurturing, that being responsive and sensitive caregiving, in order to develop optimally [1]. Maternal sensitivity is the primary predictor of long-term mother-infant attachment [2] and less secure attachment has been found in 1 to 3 years of age children born preterm compared to children born at term [3]. An increase in maternal sensitivity also predicts larger gray matter volume and head circumference in a general population of infants [4]. In children born preterm, an increase in maternal sensitivity significantly predicts improved mental development and social-emotional competence [5], more consistent and symmetric cortical thickness across brain hemispheres [6] and improved cognitive performances [7]. However, maternal sensitivity depends upon the mother’s ability to be responsive to their infant’s cues by detecting, interpreting and responding to those adequately [8], as well as the infant’s ability to demonstrate clear cues [9, 10]. According to the Synactive Theory of Development, preterm infants are able to communicate through stress and stability cues [11]. The cues widely vary from one infant to another, and the most recognizable cues for parents are the behavioral cues which include yawning, flaccidity, finger splays, etc. [11]. Yet, preterm infants lack muscle tone and physiological stability to communicate clear and evident cues, which limits their capacity to be active interaction partners with their parents [9, 12]. Thus, interventions intended to enhance maternal sensitivity, in the NICU, are indicated to enhance preterm infant neurodevelopment [10, 13–15], as preterm infants seem to benefit more from their parent’s sensitivity than infants born at term [16]. Following the emotional crisis mothers go through after having given birth prematurely [17], nurses have a central role in guiding mother to develop their sensitivity to their preterm infant’s cues in the NICU through facilitating physical closeness and participation to their infant’s care [12, 17]. In our systematic review [18] we identified 17 RCTs evaluating the effectiveness of parent-infant interventions on parental sensitivity [19–35]. Interventions evaluated in these 17 RCTs were varied but could be subdivided into two main categories being passive or active interventions. Passive interventions were defined as interventions where parents received information by healthcare professionals on different topics including (I) preterm infants’ behavioral stress and stability cues [20, 26, 28, 31], (II) available emotional support in the NICU, (III) communication and preterm infant’s needs [22], and (IV) how to support their preterm infant’s development [26]. Active interventions were defined as interventions where parents participated in an aspect of their infant’s care such as guided interventions where they were guided by an expert while interacting with or participating in their infant’s care [19, 20, 24, 25, 27, 33–35], stimulating their preterm infant’s senses [23, 29, 33], kangaroo mother care [21], and participation in their infant’s general care such as diapering and bathing [30, 32]. Meta-analyses of these interventions compared to standard care showed not statistically significant effect on parental sensitivity with high heterogeneity. However, these results are based on very low to low quality of evidence primarily because of small sample sizes and high heterogeneity showing the need for developing new and novel theory-oriented interventions to improve maternal sensitivity in the NICU [18]. Based on the paucity of good quality RCTs evaluating parent-infant interventions in the NICU, a novel intervention was developed following a theory-oriented methodology [36]. This intervention is based on the theory of guided participation [37] where mothers are guided by a nurse to interact with their preterm infant while participating in their care and therapeutic POSITioning (GP_Posit). Guided participation [38] has been used in many recent interventions that were evaluated in RCTs on the outcome of sensitivity [19, 20, 27, 33–35, 39, 40]. Moreover, positioning the preterm infant is recognised as being a central part of their care while in the NICU intended to improve their motor development [41]. One RCT evaluated the effects of a parental participation to a motor intervention for preterm infants while in the NICU [42]. Compared to standard care, results showed that preterm infants who received the motor intervention from their parents had a significantly better motor performance at term equivalent age [43], but no significant difference was found on the quality of general movements at three months of corrected age [44]. However, qualitative interviews of experimental group parents highlighted that the intervention empowered them to become competent in providing care and enhanced their feeling of attachment to their preterm infant [45]. Thus, the GP_Posit intervention, combining guided participation of mothers to their preterm infant positioning during NICU hospitalization is being pilot-tested following the below-mentioned methods and preliminary effects on maternal sensitivity and preterm infant neurodevelopment are being evaluated.

### Study objectives

#### Primary objective

The primary objective of this study is to evaluate the feasibility and acceptability of (i) the study design; and (ii) the experimental GP_Posit nursing intervention during NICU hospitalization.

#### Secondary objective

The secondary objective is to estimate the preliminary effects of GP_Posit on mothers and preterm infants’ outcomes. The specific question leading this study is : what are the preliminary effects of the GP_Posit intervention, compared to standard care, at the primary endpoint (when preterm infants will have reached 36 weeks of gestational age (WGA)) on:

- (Q1) Maternal sensitivity score to their preterm infant’s cues during oral feeding.
- (Q2) Maternal beliefs about their preterm infant’s needs and their maternal role in the NICU.
- (Q3) Preterm infant cerebral activity synchrony during eyes-closed electroencephalogram.
- (Q4) Preterm infant will have better quality of general movements.

## Methods

The methods for this pilot parallel-group RCT is presented as per the Standard Protocol Items: Recommendations for International Trials (SPIRIT) [46]. All elements of the SPIRIT checklist are reported below (also see Supplemental Material S1 for the checklist).

### Trial design

Given the paucity of evidence on intervention of the same nature as GP_Posit on similar outcomes, we chose to conduct a pilot parallel-group randomized controlled trial (RCT). This design was chosen to test the feasibility and acceptability of this complex and novel intervention in order to identify challenges that may arise prior to undertaking a large-scale RCT [47]. This trial is registered in the ClinicalTrials.gov registry (NCT03677752).

### Study setting

This study is conducted in a level III NICU of a mother and child academic hospital which is the largest NICU in Canada with a capacity of 65 newborns, accounting for approximately 129 infants born between 27 and 32 weeks of gestation each year. Moreover, these infants are cared for in single NICU rooms where parental presence is encouraged 24 h and where parents can sleep at bedside. Positioning tools, such as specialized mattresses and rolls, are available for each hospitalized infant but no specific procedure or protocol about this practice is implemented in this NICU.

### Eligibility criteria

Mother-infant dyads are recruited in the GP_Posit trial as per the following criteria. Dyads are eligible to participate if the mother (1) speaks, writes, and reads French and/or English and (2) is 18 years or older, and the preterm infant(s) is/are (3) born between 27^0/7^ and 31^6/7^ WGA, as estimated by the medical team or antenatal ultrasound performed during the first trimester, and (4) expected to be hospitalized a minimum of 4 weeks in the NICU before being transferred in another center or obtaining their discharge. These criteria have been established in order to ensure that the mother-infant dyads will be in the NICU during an acceptable period of time to participate in the intervention if randomized in the experimental group. Moreover, preterm infants born before 27^0/7^ are not included as they only start to show behavioral stress and stability cues around 28 weeks of gestational age [48] and mothers in the experimental group will be guided to detect these behavioral cues.

Dyads are not eligible if the mother (5) uses illicit substances, (6) has an unstable mental health as evaluated by a NICU healthcare professional, such as a social worker or (7) gives her infant for adoption after birth. These conditions (5 and 6) could hamper parental competencies [49, 50]. Dyads will also not be eligible if the preterm infant(s) (8) requires surgery because their health condition might be too precarious to participate in the intervention, (9) has an intraventricular hemorrhage greater than grade II, (10) has a severe genetic condition associated with neurodevelopmental impairment or (11) receives opioids or anti-epileptic drugs because these conditions might affect infants’ behavioral activity.

### Interventions

#### Experimental group: GP_Posit intervention

##### Theory supporting the experimental intervention

Mothers-infant dyads allocated to the experimental group receive the GP_Posit intervention. The intervention is primarily based on the nursing theory of guided participation (GP) [38]. Through the process of GP, the nurses providing the intervention aim to guide mothers through their care-giving practice so that they can develop appropriate parental competencies towards their preterm infant [38]. To do so, the nurses (experts) engage in a reciprocal relationship with the mothers (novices) in activities of care-giving requiring specific competencies [37, 38]. Activities can be any parental activities regarding their infant’s physical, psychological, and physiological needs as well as their infant’s health [38].

##### Experimental intervention content

The registered nurses providing the GP_Posit intervention aim to guide mothers in developing their sensitivity to their infant’s behavioral cues through positioning as a care giving activity. Most importantly, these care giving activities are only the context provided to guide mothers to interact in an appropriate matter with their infant. Consequently, the intervention nurses guide mothers in detecting their infant’s behavioral stress and stability cues throughout their participation in the care giving activities in order to develop their communication skills with their infant and sensitivity to their cues. In GP_Posit, the care giving activities primarily include basic care such as diapering followed by supine, side-lying, and prone positioning. Based on the mother’s abilities and level of confidence, these activities are being introduced by the intervention nurses in a progressive manner, throughout the weekly sessions. When the maturity of the infant allows for it, and if the mothers feel comfortable with previously introduced care giving activities, the intervention nurses will be able to add bottle-feeding or breastfeeding as an additional care-giving activity. To support the guided participation intervention, intervention nurses have access to Web-based modules developed by a multidisciplinary team at the CHU Sainte-Justine that provides written information as well as pictures and videos adapted for parents of preterm infants on various aspects of their development and care [51]. The Web-based modules were previously pilot tested by a coauthor, and results showed that parents were satisfied and that the positioning module was the most liked by parents [52]. In GP_Posit, nurses may refer to these modules when teaching mother behavioral cues and positioning techniques. Intervention nurses also give an informative booklet to experimental group mothers that contains pictures of various stress and stability cues of preterm infants so mothers can refer to it between intervention sessions.

##### Experimental intervention structure

The intervention is being provided by two neonatal registered nurses who have been trained to implement the experimental protocol. However, the same nurse always meets with the same mothers throughout the intervention. The intervention takes place at the infant’s bedside where one intervention nurse meets with the mother face to face. The intervention always takes place once a week, but the day of the week when intervention takes place depends on mother’s availabilities. The sessions start from the first week after birth to the last week before discharge or transfer to another center, or when the infant reaches 35 weeks of gestational age, whichever occurs first. It is planned that these sessions each last 30 to 45 min. During these weekly sessions, intervention nurses are demonstrating and then coaching mothers in performing the care giving activities, while always interpreting the infant’s cues and encouraging communication within the mother-infant dyad. Fathers are invited to be present during the intervention sessions, but their presence was not mandatory to be eligible to participate in the study.

#### Control group: usual care

Mother-infant dyads allocated to the control group receive usual care. In the study NICU, nurses are thought to position the preterm infants in their incubators using specialized mattresses, but parents participating in their infant’s positioning are not a current practice. Parental participation in their infant’s care is also encouraged but it is not part of a structured practice. Finally, for all infants born before 29 WGA and whose health condition has been critical, the NICU’s physiotherapists establish a positioning care plan and meet the parents to inform them about it. Otherwise, infants with plagiocephaly or torticoli are also followed by the NICU’s physiotherapist for special positioning care plans. Because of randomization, it is expected that there will be an equivalent number of infants benefiting from this particular positioning care plan in each group.

### Outcomes and data collection

Data collection starts following enrolment and consent. Data collection is performed by a member of the research team at different end points: before randomization (−*t*_1_), throughout intervention delivery (*t*_1_ through *t*_3_), and after the intervention that being when the infant reaches 36 WGA (*t*_4_).

#### Sociodemographic and clinical data

Before randomization of dyads (−*t*_1_), sociodemographic and clinical data are collected. Maternal sociodemographic data include age, origin, ethnicity, parity, marital status, level of education, prior experience of having a preterm infant or hospitalized child, type of delivery, occupation and support received by healthcare professionals such as a psychologist, social worker, lactation consultant, or psychiatrist. Sociodemographic data regarding preterm infants include sex, gestational age at birth, birth weight, and respiratory support. Clinical data is collected only for preterm infants and includes APGAR score and SNAPPE-II score [53].

#### Feasibility and acceptability outcomes

The primary objective of this study is to assess the feasibility and acceptability of (i) the study design; and (ii) the experimental GP_Posit nursing intervention during NICU hospitalization. The indicators used to evaluate the feasibility of the design are presented in Table 1 and the acceptability of the intervention in Table 2. These indicators are based upon Feeley and colleagues’ recommendations [54, 55]. A logbook where intervention nurses as well as research team members keep record of the feasibility indicators is used throughout the study (−*t*_1_ to *t*_4_). As for the acceptability indicators, mothers of both groups complete a questionnaire with yes/no questions as well as open questions at the end of their participation in the study (*t*_4_).

**Table 1:** Indicators of feasibility

Elements	Question	Indicators
**Feasibility of GP_Posit**		
Equipment and material resources	Was a computer or electronic tablet available when needed during the intervention?	Availability of computer or tablet.
	Were positioning tools available when needed during the intervention?	Availability of positioning tools.
	Is it feasible to provide mothers with an information booklet?	Number of mothers to which document was given.
Context	Is it feasible to provide GP_Posit in the individual NICU rooms?	Disruptive events.
Recruitment	Is it feasible to reach the target population?	Eligible mothers/participating mothers.
Mothers receiving the intervention	Is the intervention appropriate for mothers of preterm infants?	Mother acknowledges that it is acceptable, helpful, and relevant to participate in the intervention.
Content of the interventions	It is feasible to go through the entire planned content of GP_Posit with the mothers?	Content delivered as planned.
	Is all the necessary content already planned in GP_Posit?	Suggestions made by mothers regarding content.
	Is the content of the intervention acceptable for mothers?	Mother’s ratings regarding acceptability of the intervention.
Sequence of the intervention	Is the planned sequence of GP_Posit feasible and adequate?	Respect of the intervention sequence by intervention nurses.
Dose of the intervention	Is the planned dose of GP_Posit feasible?	Respect of the intervention dose by intervention nurses.
	Is the planned dose of GP_Posit appropriate?	Preferences of mothers in terms of the dose delivered.
Mode of intervention	Is guided participation feasible?	Intervention nurses’ opinion regarding feasibility.
	Is the utilization of online modules feasible?	Utilization of online modules as planned.
**Feasibility of the design**		
Recruitment	What is the number of dyads that were recruited compared to the number of dyads that were evaluated for eligibility?	Number of recruited mothers/mothers evaluated for eligibility.
	What are the characteristics of participating dyads?	Sociodemographic data.
	How much time is needed to reach sample size?	Time needed to reach sample size.
Randomization	Is the method of randomization feasible?	Number of non-eligible participants randomized.
Measure methods	Feasible to use EEG to measure neurodevelopment?	Number of EEGs performed.
Data collection	Is it feasible to perform data collection as planned?	% of data collection performed as planned.
Retention	Are we able to retain all dyads throughout the study?	Number of lost to follow-up and reason.
Contamination	Is there a risk of contamination?	% of mothers that were exposed to the content of the intervention.

**Table 2:** Indicators of acceptability

Elements	Question	Indicators
**Acceptability of design**		
Recruitment	Do the eligible mothers accept to participate?	% of eligible mothers that participate.
Randomisation	Do the mothers accept to be randomized?	Reason of refusal.
Data collection	Is it acceptable for mothers that we perform EEGs to their preterm infant?	80% of mothers accept.
	Is it acceptable for mothers that we perform the GMA to their preterm infant?	80% of mothers accept.
	Is it acceptable for mothers to be filmed while they breastfeed or bottle-feed?	80% of mothers accept.
	Is it acceptable for mothers to complete questionnaires?	80% of mothers accept.
General acceptability of the intervention^a^	Is it acceptable for mothers to participate in their infant’s positioning?	80% of mothers find it acceptable.
	Is it acceptable for mothers to participate in 30–45 min. intervention sessions, once to twice per week, throughout the hospitalization?	80% of mothers find it acceptable.
Appreciation^a^	Were mothers satisfied by the intervention?	80% of mothers were satisfied.
	Did mothers find it easy to participate?	80% of mothers found it easy.
	Did mothers find it challenging to participate?	Challenges encountered.
	Did the mothers find the intervention useful?	80% of mothers found it useful.
Retention^a^	Which strategies could enhance retention?	Suggested strategies by mothers.
**Acceptability of GP_Posit**		
Recruitment rate	Do eligible mothers consent to participate?	Number of eligible mothers/mothers who consent.
Attrition rate	What is the attrition rate?	Number of randomized mothers/mothers that are lost to follow-up.
**Other elements to consider**		
Contamination^a^	Do intervention mothers share content with other mothers?	Number of intervention mothers who shared content.
Co-intervention	Do mothers and/or infant participate in other research projects?	Number of mothers/infants participating in other research projects.

^a^Only for experimental group mothers

#### Other outcomes

The secondary objective of this pilot study is to estimate the preliminary effects of GP_Posit on mothers and preterm infant outcomes. Maternal outcomes include maternal sensitivity to cues and beliefs regarding their preterm infant’s needs and their own maternal role. Preterm infant outcomes include neurodevelopment.

Maternal sensitivity to cues is measured using the Parent-Child Interactions (PCI)–Feeding scale [9]. Mothers are videotaped while they feed their infant and this interaction is then coded, by a trained and certified study author who has been previously assessed for inter-rater reliability, using the 76 items of the PCI-Feeding scale. The 76 items are subdivided into four caregiver subscales (sensitivity to cues, response to distress, social-emotional growth fostering and cognitive growth fostering), and two infant subscales (clarity of cues and responsiveness to caregiver). Higher scores indicate a better sensitivity and mother-infant relationship [9]. Cronbach alphas vary from 0.6 to 0.88 and the PCI-Feeding scale has demonstrated great validity [9]. The PCI-Feeding scale has been used in at least six previous RCTs evaluating similar interventions, in the NICU, with parent-preterm infant dyads [20, 21, 23, 29, 33, 34]. Maternal beliefs about their preterm infant’s needs and their own maternal role is measured using the NICU: Parental Beliefs Scale (NICU:PBS) [56] at *t*_4_. The NICU: PBS is an auto-administered Likert type scale composed of 18 items and has a Cronbach of 0.75 to 0.91 [56].

The preterm infant’s neurodevelopment is assessed using the General Movement Assessment (GMA) tool [57] and electroencephalograms (EEG). The GMA is used to measure the quality of the newborn’s general movements repertoire [57]. A normal repertoire indicates normal neurodevelopment, and in case of neurological impairment the quality of general movements is lower. The GMA has been validated with a preterm infant population [58] and is also a valid indicator of cerebral palsy [59]. A 2-min video is taken while the infant is on its back, without any positioning aids. Videos will then be coded by a certified study author. The cerebral activity is assessed by an 8-probe portable EEG measuring resting state over a period of 30–40 min following which connectivity analyses will be performed.

#### Other confounding variables

Mother’s stress and anxiety as well as frequency of skin-to-skin contacts, parental presence at bedside and breastfeeding will be considered because of their potential to influence sensitivity [60]. The Parental Stressor Scale: Neonatal Intensive Care Unit (PSS : NICU) is used [61] to measure maternal stress. The PSS: NICU is an auto-administered Likert type scale composed of 50 items. It has been validated in a North-American context and has a Cronbach α of 0.89 to 0.94 [61]. To measure mother’s anxiety, the State-Trait Anxiety Inventory (STAI), an auto-administered questionnaire, has been selected [62]. The STAI is widely used in studies done with mothers of preterm infants in the NICU. It has been specifically developed to measure state and trait anxiety in adults with a Cronbach α of 0.93 [62]. Frequency and length of skin-to-skin contacts, frequency and length of parental presence at the infant’s bedside, and frequency of breastfeeding will be collected through medical chart review.

### Participant timeline

Mother-infant dyads are being enrolled during the first week after birth. Once informed consent is obtained, questionnaires in an opaque and sealable envelope are given to the mother to complete in the NICU room within the following 24-h after which they are sealed and collected by a member of the research team. Preterm infants also undergo the EEG, which is performed by the research team in the NICU individual room (−*t*_1_). When mothers are allocated to usual care, the nurse informs her that a member of the research team will meet with her when her infant reaches 36 WGA, or at time of discharge or transfer to another NICU, whichever occurs first. When mothers allocated to the experimental group, the intervention nurse plans the first intervention session based on the mother’s availabilities. The intervention goes on from that time until the preterm infant reaches 35 WGA (*t*_1_ through *t*_3_). To finish, a member of the research team meets with the mother when the infant reaches 36 WGA for data collection (*t*_4_). At this timepoint, the mother completes questionnaires, an EEG is performed to the preterm infant, a video of the preterm infant is taken for the GMA analysis and a feeding video is with the mother. If the preterm infant in not fully orally feeding at 36 WGA, the feeding video is done later, that being as soon as the infant is fully orally feeding. Full participant timeline is presented in Table 3.

**Table 3:**
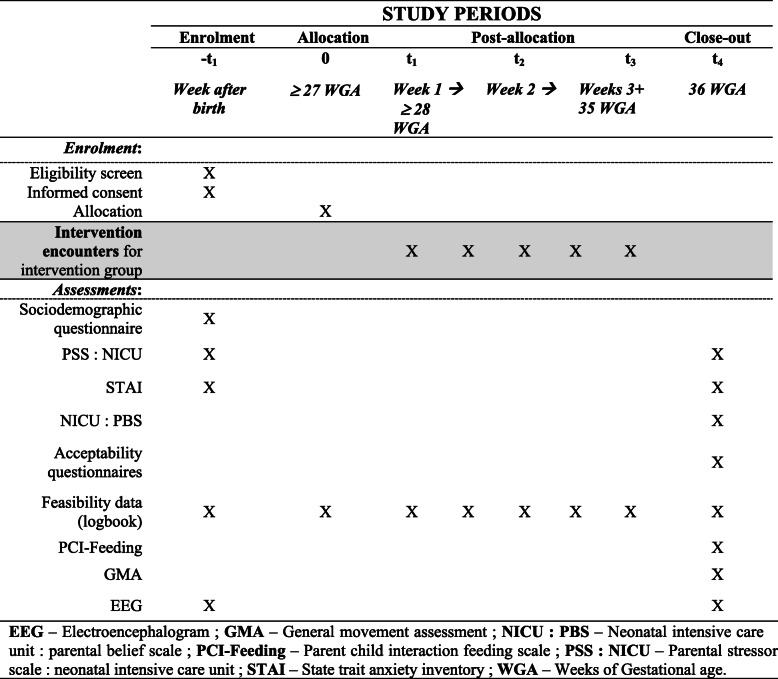
Enrolment, allocation, intervention, and data collection

### Sample size

A convenience sample of 20 mother-infant dyads will be recruited. It has been suggested that a minimum of 10 participants per group is sufficient for pilot studies [63, 64]. A maximum of 24 dyads could be recruited in case of attrition which is anticipated to be around 20% based on similar intervention studies [20, 31, 34, 35].

### Recruitment

The NICU’s research nurse presents the GP_Posit trial to each eligible mother within 1 week after having given birth. After the project has been presented, mothers can ask questions, take all the time they need to read the information form, before consenting to participate. Recruitment occurs in the postpartum unit if the mother is still hospitalized or in their preterm infant’s single NICU room.

### Randomization and allocation

An independent biostatistician of the Applied Clinical Research Unit (Unité de Recherche Clinique Appliquée (URCA)) verified the sequence of randomization as per a computer-generated random listing of interventions applying a permuted block design with random blocks of two and four. Opaque, sealed, and sequentially numbered envelopes were prepared by a professional independent from the study team. Mothers consenting to participate in the trial are randomly assigned, in a 1:1 allocation ratio, to receive either the experimental intervention or usual care. Mothers uncover their allocated group by opening the envelope in presence of the intervention nurse.

### Blinding

Due to the nature of the intervention, it is not be possible to prevent mothers and intervention nurses of knowing the dyad’s allocated group since no mock intervention is offered to the control group. Nevertheless, the research team is blinded to the mother-infant dyads’ group. Video coders will also be blinded to the dyads’ group allocation.

### Data management

An identification number is being assigned to each dyad to preserve confidentiality. All data collected will be manually entered into an electronic database statistical software, and the original questionnaires will be kept under lock and key in the principal investigator’s office at the Université de Montréal. Data entry and coding will be performed by the same person. A verification will be done by a second person to compare with the original questionnaires. Files will be maintained in storage for a period of minimum 7 years after completion of the study, according to the center’s ethical board regulation.

### Feasibility criteria

The primary criteria for assessing feasibility will be based on adhesion to the intervention which is defined as mothers participating in at least four intervention sessions with the intervention nurse. To determine feasibility of the intervention based on adhesion, at least 80% of mothers will need to have participated to a minimum of four sessions.

### Data analysis

SPSS version 25 software will be used to conduct the analyses. For all study variables, the mean and standard deviation will be presented for continuous variables, and categorical variables will be described as frequencies and percentages. No significance test will be performed because of the residual confounding that may arise from having small study groups.

Descriptive analyzes will be performed for sociodemographic and clinical variables as well as baseline maternal stress and anxiety which will be presented by study group as well as for total sample. Feasibility and acceptability variables will also be analyzed with descriptive analyses. As for maternal sensitivity to feeding cues and maternal beliefs, means will be presented by groups and effect size will be calculated using Cohen’s *d* and 95% confidence interval (CI). Regarding preterm infant neurodevelopment outcome, the GMA results will be presented as frequency of normal/abnormal for each group and the difference in proportion will be calculated. Change from baseline for maternal stress and anxiety will be compared between groups using Cohen’s *d* and 95% CI. Finally, frequency of skin-to-skin contacts, parental presence at bedside, and breastfeeding will be presented for each groups and total sample.

#### Exploratory analyses

EEG data is recorded mainly to assess the feasibility of using the 8-electrode portable device with hospitalized preterm infants as well as parent’s acceptability of performing this test for research purposes. Additional exploratory analyses will be performed on recorded EEG data. We will perform spectral analyses with multiscale entropy calculation and connectivity analyses in order to see synchrony using the phase slope index [65]. According to Bastos and Schoffelen [65], this should allow us to study the dynamic connections between neuronal populations.

### Patient and public involvement

Patients and public are not involved in the design, recruitment, and conduct of this study.

### Ethics and dissemination

Before enrolling a mother-infant dyad, informed consent is obtained from the mother for herself and her infant. Mothers are able to withdraw from the trial at any time. Confidentiality is maintained using codes to identify each mother-infant dyad. Questionnaires and videos are kept under lock to which only the first two authors have access. Ethical approval from the University Hospital ethical board was obtained in January 2018 (2017–1540).

### Protocol amendments

Since the beginning of this pilot study in late 2018, protocol amendments have been done following challenges encountered regarding recruitment. We had originally targeted a sample size of 30 (15 dyads per group) based on Julious [66] with a planned recruitment rate of two to three dyads per week. However, we initially had a recruitment rate of one dyad per month which was sub-optimal. Our solution to optimize recruitment was twofold. First, we lowered the sample size to 20 (10 dyads per group) because this sample size allowed us to estimate feasibility and acceptability. Moreover, as suggested by Leon, Davis [67], pilot studies’ sample size should be based on pragmatics such as patient flow. Second, we also made a change in inclusion criteria as we included preterm infant born between 27^0/7^ and 31^6/7^ as opposed to originally planned 28^0/7^ to 31^6/7^ WGA. Since we observed that the time needed between −*t*_1_ and *t*_1_ was of at least 1 week, preterm infants born at 27^0/7^ WGA could participate as they would be 28^0/7^ WGA by the time the experimental intervention would start.

### Trial status

This study is currently ongoing.

## Discussion

This pilot RCT aims at evaluating the feasibility and acceptability of an innovative and complex nursing intervention combining positioning of preterm infants with the participation of their mothers. This is one of few studies evaluating an intervention combining maternal participation to developmental care (positioning). Similar interventions have shown significant effects of the experimental intervention on parental sensitivity [21, 24, 27, 28, 30, 33]. The intervention is also well fitted with the paradigm shift in relation to the reconfiguration of the single family NICU rooms and is coherent with the individualization of care and empowerment of parents as soon as the hospitalization in the NICU. This research has also the potential to identify a new intervention which may promote parental sensitivity and preterm infant neurodevelopment and thus, long term quality of life for these families. However, evaluating a complex intervention may be challenging because various aspects of the implementation process may not be suitable for the population and/or setting. Thus, conducting a pilot study is relevant because it will allow to identify areas for improvement about the methods or intervention before a large-scale RCT. In fact, the early stages of recruitment already allowed us to identify difficulties in recruiting. Amendments to the protocol were brought and the recruitment rate increased.

## Supplementary information

**Additional file 1.**

## Data Availability

Not applicable.

## Abbreviations

URCA: Unité de Recherche Clinique Appliquée
EEG: Electroencephalogram
GMA: General Movement Assessment
GP: Guided participation
GP_Posit: Guided participation in care and positioning
NICU: Neonatal intensive care unit
NICU: PBS: NICU: Parental Beliefs Scale
PSS : NICU: Parental Stressor Scale: Neonatal Intensive Care Unit
PCI: Parent-child interaction
RCT: Randomized controlled trial
STAI: State-Trait Anxiety Inventory
WGA: Weeks of gestational age

## References

[1] DeMaster D, Bick J, Johnson U, Montroy JJ, Landry S, Duncan AF (2019). Nurturing the preterm infant brain: leveraging neuroplasticity to improve neurobehavioral outcomes. Pediatr Res..

[2] Deans Carolyn L. (2018). Maternal sensitivity, its relationship with child outcomes, and interventions that address it: a systematic literature review. Early Child Development and Care.

[3] Ruiz N, Piskernik B, Witting A, Fuiko R, Ahnert L (2018). Parent-child attachment in children born preterm and at term: A multigroup analysis. PLoS One..

[4] Kok R, Thijssen S, Bakermans-Kranenburg MJ, Jaddoe VW, Verhulst FC, White T (2015). Normal variation in early parental sensitivity predicts child structural brain development. J Am Acad Child Adolesc Psychiatry..

[5] Treyvaud K, Anderson VA, Howard K, Bear M, Hunt RW, Doyle LW (2009). Parenting behavior is associated with the early neurobehavioral development of very preterm children. Pediatrics.

[6] Frye RE, Malmberg B, Swank P, Smith K, Landry S (2010). Preterm birth and maternal responsiveness during childhood are associated with brain morphology in adolescence. J Int Neuropsychol Soc..

[7] Banerjee N (2018). Are maternal depression, breastfeeding, matenal alcohol intake, and infant biological vulnerability, effect modifiers of confounders of the maternal sensitivity-cognitive development association?.

[8] Ainsworth MD, Blehar MC, Waters E, Wall SN. Patterns of Attachment: A Psychological Study of the Strange Situation: Erlbaum, Hillside; 1978.

[9] Oxford M, Findlay D (2015). NCAST Caregiver / Parent-Child interaction Feeding Manual.

[10] Neuhauser A (2016). Predictors of maternal sensitivity in at-risk families. Early Child Development Care..

[11] Als H (1982). Toward a Synactive Theory of Development: promise for the assessment and support of infant individuality. Infant Ment Health J..

[12] Fleck P (2016). Connecting mothers and infants in the neonatal intensive care unit. Newborn Infant Nurs Rev..

[13] Bilgin A, Wolke D (2015). Maternal sensitivity in parenting preterm children: a meta-analysis. Pediatrics..

[14] Milgrom J, Newnham C, Anderson PJ, Doyle LW, Gemmill AW, Lee K (2010). Early sensitivity training for parents of preterm infants: impact on the developing brain. Pediatr Res..

[15] Poehlmann J, Hane A, Burnson C, Maleck S, Hamburger E, Shah PE (2012). Preterm infants who are prone to distress: differential effects of parenting on 36-month behavioral and cognitive outcomes. J Child Psychol Psychiatry..

[16] Jaekel J, Pluess M, Belsky J, Wolke D (2015). Effects of maternal sensitivity on low birth weight children's academic achievement: a test of differential susceptibility versus diathesis stress. J Child Psychol Psychiatry..

[17] Fernandez Medina IM, Granero-Molina J, Fernandez-Sola C, Hernandez-Padilla JM, Camacho Avila M, Lopez Rodriguez MDM (2018). Bonding in neonatal intensive care units: Experiences of extremely preterm infants' mothers. Women Birth..

[18] Lavallee A, Aita M, Bourbonnais A, et al. Effectiveness of early interventions for parental sensitivity following preterm birth: a systematic review protocol. Syst Rev. 2017;6(62):e1–5. 10.1186/s13643-017-0459-x. 10.1186/s13643-017-0459-x PMC5364600 28335806

[19] Borghini A, Habersaat S, Forcada-Guex M, Nessi J, Pierrehumbert B, Ansermet F (2014). Effects of an early intervention on maternal post-traumatic stress symptoms and the quality of mother-infant interaction: the case of preterm birth. Infant Behav Dev..

[20] Browne JV, Talmi A (2005). Family-based intervention to enhance infant-parent relationships in the neonatal intensive care unit. J Pediatr Psychol..

[21] Chiu SH, Anderson GC (2009). Effect of early skin-to-skin contact on mother-preterm infant interaction through 18 months: randomized controlled trial. Int J Nurs Stud..

[22] Evans T, Boyd RN, Colditz P, Sanders M, Whittingham K (2017). Mother-very preterm infant relationship quality: RCT of baby triple P. J Child Family Stud.

[23] Glazebrook C, Marlow N, Israel C, Croudace T, Johnson S, White IR (2007). Randomised trial of a parenting intervention during neonatal intensive care. Archives Dis Childhood Fetal Neonatal Edition..

[24] Hane A, Myers MM, Hofer MA, Ludwig RJ, Halperin MS, Austin J (2015). Family nurture intervention improves the quality of maternal caregiving in the neonatal intensive care unit: Evidence from a Randomized Controlled Trial. J Dev Behav Pediatr..

[25] Hoffenkamp HN, Tooten A, Hall RA, Braeken J, Eliens MP, Vingerhoets AJ (2015). Effectiveness of hospital-based video interaction guidance on parental interactive behavior, bonding, and stress after preterm birth: a randomized controlled trial. J Consult Clin Psychol..

[26] Melnyk BM, Feinstein NF, Alpert-Gillis L, Fairbanks E, Crean HF, Sinkin RA (2006). Reducing premature infants' length of stay and improving parents' mental health outcomes with the Creating Opportunities for Parent Empowerment (COPE) neonatal intensive care unit program: a randomized, controlled trial. Pediatrics..

[27] Meyer EC, Coll CT, Lester BM, Boukydis CF, McDonough SM, Oh W (1994). Family-based intervention improves maternal psychological well-being and feeding interaction of preterm infants. Pediatrics..

[28] Milgrom J, Newnham C, Martin PR, Anderson PJ, Doyle LW, Hunt RW (2013). Early communication in preterm infants following intervention in the NICU. Early Hum Dev..

[29] Nelson MN, White-Traut RC, Vasan U, Silvestri J, Comiskey E, Meleedy-Rey P (2001). One-year outcome of auditory-tactile-visual-vestibular intervention in the neonatal intensive care unit: effects of severe prematurity and central nervous system injury. J Child Neurol..

[30] Newnham CA, Milgrom J, Skouteris H (2009). Effectiveness of a modified Mother-Infant Transaction Program on outcomes for preterm infants from 3 to 24 months of age. Infant Behavior Dev.

[31] Ravn IH, Smith L, Lindemann R, Smeby NA, Kyno NM, Bunch EH (2011). Effect of early intervention on social interaction between mothers and preterm infants at 12 months of age: a randomized controlled trial. Infant Behavior and Dev.

[32] Teti DM, Black MM, Viscardi R, Glass P, O’Connell MA, Baker L (2009). Intervention With African American Premature Infants: Four-Month Results of an Early Intervention Program. J Early Intervention..

[33] White-Traut RC, Nelson MN (1988). Maternally administered tactile, auditory, visual, and vestibular stimulation: relationship to later interactions between mothers and premature infants. Res Nurs Health.

[34] White-Traut R, Norr KF, Fabiyi C, Rankin KM, Li Z, Liu L (2013). Mother-infant interaction improves with a developmental intervention for mother-preterm infant dyads. Infant Behavior Dev.

[35] Zelkowitz P, Feeley N, Shrier I, Stremler R, Westreich R, Dunkley D (2011). The cues and care randomized controlled trial of a neonatal intensive care unit intervention: effects on maternal psychological distress and mother-infant interaction. J Dev Behav Pediatr..

[36] Craig P, Dieppe P, Macintyre S, Michie S, Nazareth I, Petticrew M (2008). Developing and evaluating complex interventions: the new Medical Research Council guidance. BMJ..

[37] Schroeder M, Pridham KF (2006). Development of relationship competencies through guided participation for mothers of preterm infants. J Obstetric Gynecologic Neonatal Nurs.

[38] Pridham KF, Limbo R, Schroeder M, Thoyre S, Van Riper M (1998). Guided participation and development of care-giving competencies for families of low birth-weight infants. J Adv Nurs..

[39] Twohig A, Segurado R, McCarthy A, Underdown A, McNicholas F, Molloy EJ (2019). Early intervention to support preterm infant-parent interaction and development: results of a randomised controlled trial on maternal sensitivity, social-emotional development and parental mental health. Arch Dis Child..

[40] Xie J, Zhu L, Zhu T, Jian Y, Ding Y, Zhou M (2019). Parental Engagement and Early Interactions With Preterm Infants Reduce Risk of Late Postpartum Depression. J Nerv Ment Dis..

[41] Lavallée A, De Clifford-Faugere G, Matte C, Aita M (2018). Effets bénéfiques du positionnement sur le développement du nouveau-né prématuré. Cahiers de la Puéricultrice..

[42] Oberg GK, Campbell SK, Girolami GL, et al. Study protocol: an early intervention program to improve motor outcome in preterm infants: a randomized controlled trial and a qualitative study of physiotherapy performance and parental experiences. BMC Pediatr. 2012;12(15):e1–9. 10.1186/1471-2431-12-15. 10.1186/1471-2431-12-15 PMC3305610 22336194

[43] Ustad T, Evensen KAI, Campbell SK, Girolami GL, Helbostad J, Jørgensen L (2016). Early Parent-Administered Physical Therapy for Preterm Infants: A Randomized Controlled Trial. Pediatrics..

[44] Fjørtoft T, Ustad T, Follestad T, Kaaresen PI, Øberg GK (2017). Does a parent-administrated early motor intervention influence general movements and movement character at 3 months of age in infants born preterm?. Early Hum Dev..

[45] Øberg GK, Ustad T, Jørgensen L, Kaaresen PI, Labori C, Girolami GL (2018). Parents’ perceptions of administering a motor intervention with their preterm infant in the NICU. Eur J Physiotherapy..

[46] Chan AW, Tetzlaff JM, Altman DG, Laupacis A, Gotzsche PC, Krleza-Jeric K (2013). SPIRIT 2013 statement: defining standard protocol items for clinical trials. Ann Intern Med..

[47] Craig P, Dieppe P, Macintyre S, Michie S, Nazareth I, Petticrew M (2013). Developing and evaluating complex interventions: the new Medical Research Council guidance. Int J Nurs Stud..

[48] Fern D (2011). A neurodevelopmental care guide to positioning & handling the premature, fragile or sick infant.

[49] Neger EN, Prinz RJ (2015). Interventions to address parenting and parental substance abuse: conceptual and methodological considerations. Clin Psychol Rev..

[50] Stein A, Pearson RM, Goodman SH, Rapa E, Rahman A, McCallum M (2014). Effects of perinatal mental disorders on the fetus and child. Lancet..

[51] Luu TM, Gosselin J, Karsenti T, Côté S, Walker DC, Peckre P, et al. Mieux Agir au Quotidien: Comprendre et soutenir le développement de mon enfant. 2015. Available from: http://developpementenfant.ca/wp/. Accessed Feb 2020.

[52] Luu TM, Xie LF, Peckre P, Cote S, Karsenti T, Walker CD (2017). Web-based intervention to teach developmentally supportive care to parents of preterm infants: feasibility and acceptability study. JMIR Res Protoc..

[53] Harsha SS, Archana BR (2015). SNAPPE-II (Score for Neonatal Acute Physiology with Perinatal Extension-II) in Predicting Mortality and Morbidity in NICU. J Clin Diagn Res..

[54] Feeley N, Cossette S, Henly SJ (2016). Pilot studies for randomized clinical trials. The Routledge International Handbook of Advanced Quantitative Methods in Nursing Research.

[55] Feeley N, Cossette S, Cote J, Heon M, Stremler R, Martorella G (2009). The importance of piloting an RCT intervention. Can J Nurs Res..

[56] Melnyk BM, Oswalt KL, Sidora-Arcoleo K (2014). Validation and psychometric properties of the neonatal intensive care unit parental beliefs scale. Nurs Res..

[57] Einspieler C, Prechtl HF (2005). Prechtl's assessment of general movements: a diagnostic tool for the functional assessment of the young nervous system. Ment Retard Dev Disabil Res Rev..

[58] Prechtl HF (2001). General movement assessment as a method of developmental neurology: new paradigms and their consequences. Dev Med Child Neurol..

[59] Adde L, Rygg M, Lossius K, Oberg GK, Stoen R (2007). General movement assessment: predicting cerebral palsy in clinical practise. Early Hum Dev..

[60] Shin H, Park YJ, Ryu H, Seomun GA (2008). Maternal sensitivity: a concept analysis. J Adv Nurs..

[61] Miles MS, Funk SG, Carlson J (1993). Parental Stressor Scale: neonatal intensive care unit. Nurs Res.

[62] Spielberger CD, Gorsuch RL, Lushene R, Vagg PR, Jacobs GA (1983). State-trait anxiety inventory for adults.

[63] Moore CG, Carter RE, Nietert PJ, Stewart PW (2011). Recommendations for planning pilot studies in clinical and translational research. Clin Transl Sci..

[64] Hertzog MA (2008). Considerations in determining sample size for pilot studies. Res Nurs Health..

[65] Bastos AM, Schoffelen JM (2015). A tutorial review of functional connectivity analysis methods and their interpretational pitfalls. Front Syst Neurosci..

[66] Julious SA (2005). Sample size of 12 per group rule of thumb for a pilot study. Pharm Stat..

[67] Leon AC, Davis LL, Kraemer HC (2011). The role and interpretation of pilot studies in clinical research. J Psychiatr Res..

